# Dr. Adams, on the Slough

**Published:** 1805-05-01

**Authors:** Joseph Adams


### To Dr. BRADLEY.

DEAR SIR,

WHEN I last addressed you on the subject of Vaccination, and on the true diagnosis of Small-pox, I did it with a degree of confidence which many of your readers may think rash. However, as I produced all my authorities, I hope that circumstance will be considered, if not a sufficient ficient apology, at least a fair introduction to my present confession. I first was induced to search for the slough, which lines the foveolus in small-pox by Mr. Hunter's paper. Astonished at the facility with which it presented itself, it became my amusement to exhibit it, whenever I met with the disease. At the S. P. Hospital, I discovered and exhibited it to the surgeons of that charity in every case I examined. In private practice I shewed it to Mr. Pitcairn. On my return to England I prevailed on Mr. King, of Clifton, to look for it, who had no more difficulty in detecting it than myself; and before I used the strong language with which my last concludes, I conversed with Dr. Willan, who confirmed me in a presumption which was strong enough before.

With all this confidence, nothing seemed to remain but an opportunity, by the return of the disease, of exhibiting the slough to such as would have the best means of communicating this diagnosis. Dr. Hooper has favoured me with this opportunity; but, to my great mortification, I could not exhibit the slough. However, on a very slight examination of the subject, I found he had that species of small-pox which used to be called christalline, and of which, on a former occasion, I expressed my doubts whether they contained the slough.* But my mortification was not to end here. Dr. Hooper had other subjects for my inquiry, and my confidence was not lessened by the recent event. However, disappointment still pursued me. Though the bottoms of the foveoli were sloughy, I could not take up with the forceps any thing that resembled a piece of soaked paper. I have since tried it in other instances, but have been equally disappointed; insomuch that, had I not been able to produce such a number of witnesses, or had the discovery commenced with myself, I might have been induced to doubt the conviction of my own senses. As it is, I thought it my duty to be thus early in correcting my own errors, and to remind all the profession that there are still minutiæ in small-pox not perfectly understood. The discovery of the cow-pox has done much toward an accurate inquiry on these subjects, and will ultimately, no doubt, render all inquiries of the kind superfluous. But I hope, in the mean while, your readers will be diligent in marking, not only general appearances in eruptions, but in dissecting them, and recording with their description a minute history of the symptoms in their order.

Your's, &c.

JOSEPH ADAMS.

* See Journal, vol. ix. p. 312.

